# Neuroprotective Strategy in Retinal Degeneration: Suppressing ER Stress-Induced Cell Death via Inhibition of the mTOR Signal

**DOI:** 10.3390/ijms18010201

**Published:** 2017-01-19

**Authors:** Bin Fan, Ying-Jian Sun, Shu-Yan Liu, Lin Che, Guang-Yu Li

**Affiliations:** Department of Ophthalmology, Second Hospital of Jilin University, Changchun 130041, China; fanbins@aliyun.com (B.F.); Sunyj16@mails.jlu.edu.cn (Y.-J.S.); Shuyan16@mails.jlu.edu.cn (S.-Y.L.); Chelin16@mails.jlu.edu.cn (L.C.)

**Keywords:** mTOR, ER stress, retinal degeneration, unfolded protein response, retinal neuroprotection, apoptosis

## Abstract

The retina is a specialized sensory organ, which is essential for light detection and visual formation in the human eye. Inherited retinal degenerations are a heterogeneous group of eye diseases that can eventually cause permanent vision loss. UPR (unfolded protein response) and ER (endoplasmic reticulum) stress plays an important role in the pathological mechanism of retinal degenerative diseases. mTOR (the mammalian target of rapamycin) kinase, as a signaling hub, controls many cellular processes, covering protein synthesis, RNA translation, ER stress, and apoptosis. Here, the hypothesis that inhibition of mTOR signaling suppresses ER stress-induced cell death in retinal degenerative disorders is discussed. This review surveys knowledge of the influence of mTOR signaling on ER stress arising from misfolded proteins and genetic mutations in retinal degenerative diseases and highlights potential neuroprotective strategies for treatment and therapeutic implications.

## 1. Retinal Degeneration

The retina arises from the neuroectoderm during embryogenesis and is the part of the eye that perceives light stimuli, as well as integrates and transmits electrical impulses through the optic nerve to the visual cortex in the brain. The retina is comprised of six major types of neurons, including retinal ganglion cells, bipolar, horizontal and amacrine interneurons, Müller glia and photoreceptors. These neurons are organized in three cellular layers separated by synaptic layers [1]. In the outer nuclear layer (ONL), the photoreceptor cells are morphologically compartmentalized cells having inner and outer segment regions, connected by a narrow cilium. The outer segment of the photoreceptor consists of a stack of disk membranes surrounded by a plasma membrane [2]. Continual replenishment of disks in the photoreceptor leads to a high rate of protein turnover and ER biogenesis post-translationally modifies and controls the quality of many outer segment (OS) proteins, including opsin. Retinal pigment epithelial (RPE) cells, located at the outer layer of the retina, provide nourishment (e.g., vitamin A metabolites) and clear OS debris of the overlying photoreceptor cells, via daily phagocytosis of OS tips, and participate in regeneration of visual pigment regeneration [3]. The outer plexiform layer (OPL) consists of synaptic interactions between photoreceptors and horizontal cells and bipolar cells [4]. The inner nuclear layer (INL) contains horizontal, bipolar, and amacrine cell bodies, which play different roles in visual formation. The inner plexiform layer (IPL) is composed of synapses between bipolar and retinal ganglion cells (RGCs), and the ganglion cell layer (GCL) consists of RGC nuclei. The RGC axons form the optic nerve from the output of the retina to the brain, transferring visual information to the centers [5].

Inherited retinal degenerations (IRD) are a heterogeneous group of eye diseases, which affect more than 2 million people worldwide, that can eventually cause permanent vision loss [6]. The inheritance patterns, onset age, and severity of visual dysfunction in IRD are different. Syndromic and nonsyndromic forms of retinal dystrophies, which include autosomal, X-linked, and mitochondrial inheritance are classified. Phenotypic categories cover retinitis pigmentosa (RP), macular degeneration, cone or cone-rod dystrophy, congenital stationary night blindness, and Leber congenital amaurosis (LCA) etc. [7]. Dysfunctions of rod and cone photoreceptor cells, can involve the outer segment structure, phototransduction, the cilium structure and transport connection, inner segment protein and vesicle trafficking, lipid metabolism, chaperone function, RNA splicing and transcription, synaptic function, and retinal development. Affected mechanisms in the RPE cover membrane trafficking, ion transport and visual cycle reactions [7]. Certain mutations in secondary retinal neurons such as ganglion cells and Müller cellscan also lead to IRD [8]. However, the exact molecular mechanisms involved in mutant genes causing dysfunctional retinal neurons to undergo apoptosis and lead to the development of IRDs are still unclear. Animal models, which carry mutations in various genes that mimic human IRDs have been used to describe the modes of retinal cell death. These animals include (1) retinal degeneration (rd) mice; (2) retinal degeneration slow (rds) mice; (3) transgenic mice carrying P347S and Q344ter mutations in the rhodopsin gene; (4) knockout mice deficient for the b2-subunit of Na1/K1-ATPase expressed in retinal Müller cells; and (5) Royal College of Surgeons (RCS) rats, in which photoreceptors were detected that diedvia the apoptotic pathway as evidenced by histological morphology, TUNEL (terminal deoxynucleotidyl transferase-mediated biotin-dUTP nick end-labeling) assays, and/or by retinal DNA laddering with gel electrophoresis. Results from these studies indicate that it is likely that photoreceptors in human IRDs are dying similarly via the apoptotic pathway [9].

## 2. Proteostasis and ER Stress

Protein homeostasis is critical for cellular function andi s tightly controlled by the synthesis and clearance of proteins [10]. The concept of proteostasis is simple. Protein synthesis (including protein folding and protein transport) must match the rate of degradation [11,12]. A healthy proteomeis maintained through a series of complex surveillance systems, which ensure that each protein is functionally folded or assembled [13]. Cells have evolved many mechanisms to cope with misfolded proteins, such as the ubiquitin-proteasome system (UPS) [14], ER-associated protein degradation (ERAD) [15] and the unfolded protein response (UPR) [16]. These proteostasis networks play important roles in maintaining correctly folded proteins and removing misfolded proteins.

The endoplasmic reticulum (ER) is responsible for the quality control of newly synthesized proteins, including protein folding, post-translational modification and transportation [17], thus it is a key component of cellular proteostasis. ER cisternae have been historically classified as ribosome-bound “rough” ER and ribosome-free “smooth” ER. As indicated, smooth ER includes ribosome-free areas, where fusion and vesicle budding take place, whereas the rough ER performs the functions of proper protein folding and modification [18]. The newly synthesized, unfolded proteins are initially generated from the ribosomes and are then transported into the cisternal space of the ER, where these polypeptide chains are properly folded and oligomerized, disulfide bonds are formed, and *N*-linked oligosaccharides are attached for a glycoprotein. After folding and post-translational modification, mature proteins are disassociated from ER chaperones and transported to the Golgi apparatus [19].

**Figure 1:**
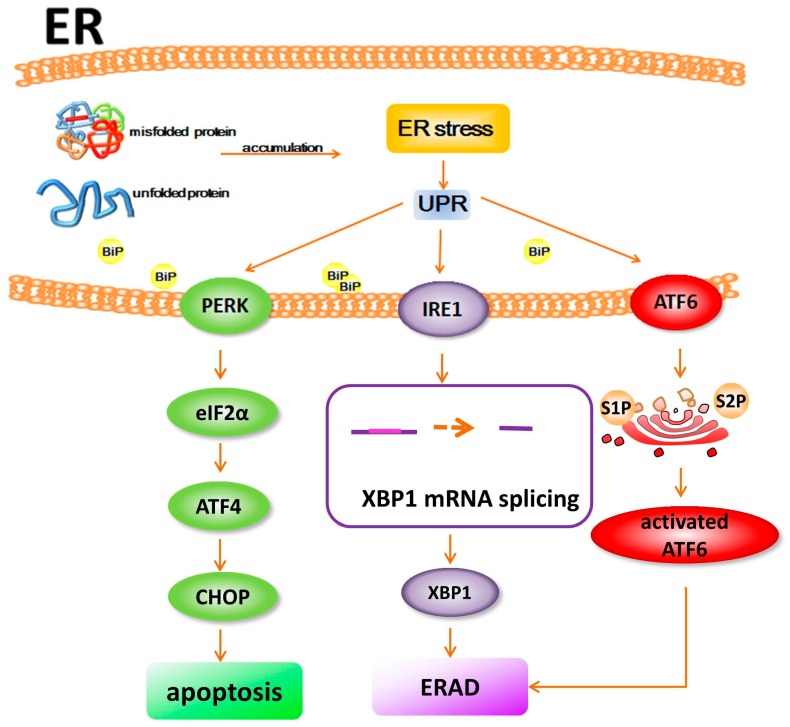
The unfolded protein response (UPR).There are three known pathways triggering UPR in ER. (i) PERK (protein kinase RNA-like ER kinase) phosphorylates initiation factor eIF2α, leading to cap independent translation of ATF4 (activating transcription factor 4). ATF4 activates CHOP (C/EBP homologous protein), which can stimulate apoptosis; (ii) IRE1 (inositol-requiring protein 1), is a kinase that leads to activation of RNAse activity. This induces the splicing of XBP1 mRNA and further activates ERAD (ER-associated protein degradation); (iii) following BiP (Immunoglobulin binding protein) dissociation, ATF6 is cleaved by the S1P and S2P proteases into an active form in the Golgi. The activated ATF6 will then cause the activation of ERAD to restore ER (endoplasmic reticulum) homeostasis.

The cellular protein folding capacity is tightly regulated in the ER via activation of intracellular signal pathways [20]. When the accumulation of misfolded or unfolded proteins causes an imbalance in ER homeostasis, it leads to ER stress, which further causes the activation of the UPR (unfolded protein response) [21,22]. The UPR promotes protein folding and suppresses protein translation to reduce the load of proteins within the ER and increases autophagy and ERAD to promote degradation of misfolded proteins [21]. There are three known stress sensors that trigger UPR in ER: inositol-requiring protein 1 (IRE1), protein kinase RNA-like ER kinase (PERK), and activating transcription factor 6 (ATF6) [23]. PERK phosphorylates initiation factor eIF2α, leading to cap independent translation of ATF4. ATF4 activates C/EBP homologous protein (CHOP), which can stimulate apoptosis. IRE1 is a kinase that leads to activation of RNAse activity. This induces the splicing of XBP1mRNA and further activates ERAD. Following immunoglobulin binding protein (BiP) dissociation, ATF6 is cleaved by the S1P and S2P proteases into an active form in the Golgi. The activated ATF6 then causes activation of ERAD to restore ER homeostasis. The initial transcriptional and translational effects of IRE1, PERK, and ATF6 signaling help cells adapt to ER stress. However, if these actions fail to restore ER homeostasis and ER stress persists, UPR signaling triggers maladaptive proapoptotic programs and cell death. ER stress functions as a critical mechanism relevant to pathogenesis in IRD [19] (Figure 1).

## 3. Disturbance of Proteostasis and ER Stress in Retinal Degeneration

The generation of visual information from retinal cells depends on functional proteins, such as rhodopsin (Rh) [24]. ER is an important intracellular apparatus responsible for the protein quality control. Only properly folded proteins are released from the ER, yet misfolded proteins are degraded to prevent the generation of dysfunctional or potentially toxic proteins [25]. The ER stress and UPR, caused by protein misfolding, have been recently regarded as a contributing factor to IRD [26,27]. Bhootada et al. reported that the expression of T17M Rh in rod photoreceptors induces the activation of ER stress-related UPR signaling, which results in severe retinal degeneration. ATF4 knockdown blocked retinal degeneration and promoted photoreceptor survival in one-month-old T17M mice [28]. Thus far, over 250 different heritable mutations have been identified that cause the production of abnormal proteins by retinal cells, which lead to retinal degeneration and vision loss [29]. The imbalance of proteostasis has been implicated in IRD [30,31]. UPR induction might function as a common pathway, which is activated in cases of retinal degeneration, involving the degenerative process.

As the predominant protein within photoreceptors, mutations in Rh are the most common cause of inherited RP [32]. The IRE1 signaling pathway of UPR was robustly activated in a *Drosophila* model of retinal degeneration caused by Rh misfolding [33]. In addition, selective activation of UPR signaling pathways were detected in P23H animals, which is expressed at different levels of P23H Rh compared to wild-type siblings [34,35,36,37]. Griciuc et al. studied *Drosophila*, in which Rh1 (P37H) was expressed in photoreceptors, and genetically increased the levels of misfolded Rh1 (P37H), which further caused the activation of the Ire1/Xbp1 ER stress pathway [38]. In animals expressing misfolded Rh, proapoptotic UPR molecules, such as cleaved ATF6, pEIF2, and CHOP, markedly increased before the loss of photoreceptors, raising the possibility that UPR activation caused by misfolded Rh may directly result in photoreceptor cell death [34,35]. However, overexpressing BiP with adeno-associated virus type 5 (AAV5) vector in transgenic rat retina led to a reduction in CHOP and photoreceptor apoptosis [35].

The mutations in the *ELOVL4* (the elongation of very long chain fatty acids) gene resulted in Stargardt macular dystrophy and early childhood blindness [39]. *ELOVL4* encodes a membrane protein targeted to the ER, which is an enzyme involved in the generation of long-chain fatty acids [40,41]. Not only do photoreceptors express high levels of *ELOVL4*, but other types of cells in the eye have been found to express *ELOVL4* as well [42]. From a molecular point of view, *ELOVL4* mutations cause premature truncations of the protein, leading to loss of an ER retention motif [43,44,45]. In transfected cells, misfolded *ELOVL4* aggregates in the ER and induces the UPR, which is closely associated with photoreceptor cell death [35].

The rd1 mutation leads to a remarkable reduction in PDE6-β protein, which is a catalytic subunit of a phosphodiesterase, regulating cGMP levels in photoreceptors [46,47]. Absence of the *PDE6-β* causes accumulation of cGMP and, in turn, results in significant increases of intracellular calcium and photoreceptor degeneration [48]. In a recent study it was demonstrated that multiple UPR signaling pathways were activated and UPR protein levels of BiP and peIF2α were increased in a time-dependent manner in the retinas of rd1 mice, which suggests that the ER stress contributes to retinal degeneration in rd1 mice [49].

As a post-translational modification, *N*-linked glycosylation can influence protein folding efficiency. The fibulin-3 gene encodes an *N*-glycoprotein, which is expressed and secreted by RPE cells. The *R345W* mutation leads to fibulin-3 misfolding and retention in the ER and causes Malattia Leventinese and Doyne honeycomb retinal dystrophy (ML/DHRD) [50,51,52]. In a cell study, over-expression of mutant *R345W* fibulin-3 resulted in activation of UPR and increased vascular endothelial growth factor (VEGF) expression when compared with over expression of the wild-type protein [50,53]. These results suggest that misfolded fibulin-3 may trigger RPE dysfunction via activation of UPR pathways, yet further enhance the VEGF level leading to choroidal neovascularization.

Various gene mutations target different retinal neurons and encode misfolded proteins in various types of retinal degenerative diseases. As misfolded or unfolded proteins accumulate in the ER, the UPR is triggered to restore proteostasis. However, an excessive imbalance in proteostasis results in prolonged ER stress, which initiates programmed cell death. The ER stress response has been recently proposed as an important contributing factor to retinal degenerative disease (Table 1).

## 4. mTOR and mTOR Signal

The mTOR (the mammalian target of rapamycin) is an evolutionarily-conserved serine/threonine kinase, which belongs to PIKK (the PI3K-kinase-related kinase) superfamily [19,56]. mTOR functions as two physically and functionally distinct signaling complexes, mTOR complex 1 and 2 (mTORC1 and mTORC2) [57]. mTORC1 is composed of RAPTOR (regulatory associated protein of mTOR), PRAS40 (40 kDa pro-rich AKT1 substrate 1), mLST8 (mammalian lethal with SEC13 protein 8), and DEPTOR (DEP domain-containing mTOR-interacting protein). mTORC2 consists of RICTOR (rapamycin-insensitive companion of mTOR), PRR5 (pro-rich protein 5), mSIN1 (stress-activated MAP kinase-interacting protein 1), mLST8, and DEPTOR [58]. mTORC1 is sensitive to rapamycin, yet mTORC2 is not directly inhibited by rapamycin.

**Figure 2:**
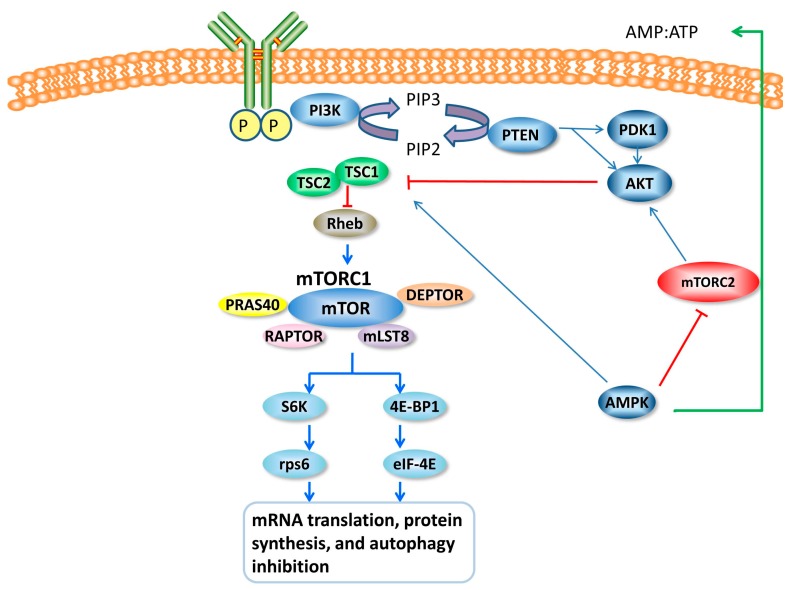
Schematic representation of the mTOR signaling pathway. mTORC1 is activated by Rheb (Ras homolog enriched in brain); the two most well-known downstream targets of mTORC1 are S6K1 (S6 kinase 1) and 4EBP1 (eukaryotic translation initiation factor 4E-binding protein 1). As an upstream signal, TSC (tuberous sclerosis complex) can suppress Rheb to negatively regulate mTORC1. Furthermore, PTEN (phosphatase and tensin homolog deleted on chromosome 10) can be suppressed by PI3K (phosphoinositide 3-kinase), thus inactivating the mTOR pathway. In addition, AMPK (AMP-dependent kinase) is activated by a high AMP/ATP ratio and suppresses mTOR. The main function of mTORC1 activity is the stimulation of mRNA translation, protein synthesis, and autophagy inhibition. PIP2/PIP3, Phosphatidylinositol 4,5-bisphosphate/Phosphatidylinositol 3,4,5-triphosphate; PDK1, 3-Phosphoinositide-dependent protein kinase 1; AKT1, AKT serine/threonine kinase 1; PRAS40, 40 kDa pro-rich AKT1 substrate 1; RAPTOR, regulatory associated protein of mTOR; mLST8, mammalian lethal with SEC13 protein 8; DEPTOR, DEP domain-containing mTOR-interacting protein.

mTORC1 is potently activated by a small GTPase known as Rheb (Ras homolog enriched in brain), yet Rhebcanbe is suppressed by TSC (tuberous sclerosis complex), which consists of TSC1 and TSC2 [59]. In addition, AKT also activates the mTOR signal directly by inhibiting PRAS40 [60]. PTEN (phosphatase and tensin homolog deleted on chromosome 10), which is further upstream, can be suppressed PI3K, thus inactivating the mTOR pathway [61]. In addition, functioning as a key sensor of relative energy status, AMPK (AMP-dependent kinase) is activated by a high AMP/ATP ratio and suppresses mTOR in the cell as an upstream signal [62]. The two most well-known downstream targets of mTORC1 are S6K1 (S6 kinase 1) and 4EBP1 (eukaryotic translation initiation factor 4E-binding protein 1). The main function of mTORC1 activity is the stimulation of mRNA translation, protein synthesis, and autophagy inhibition [62] (Figure 2).

## 5. Inhibition of mTOR Suppresses ER Stress and Attenuates Retinal Degeneration

Rapamycin (a.k.a. *Rapa Nui*), as an mTOR inhibitor, was discovered in 1970 on Easter Island. In all eukaryotes, the intracellular rapamycin receptor is a 12-kDa protein, the FK506-binding protein (FKBP12) [63]. When rapamycin conjugates to FKBP12, it forms a ternary complex with a conserved mTOR domain, shutting down downstream signals [64,65].

Rapamycin inhibits the function of mTORC1, whereas newly-developed mTOR kinase inhibitors interfere with the actions of both mTORC1 and mTORC2. mTOR inhibition suppresses cellular protein synthesis by regulating the initiation and elongation phases of translation and ribosome biosynthesis [66]. Two main downstream signals of mTOR are p70S6K and eIF4E (4E-BP1) [67]. Blocking mTOR affects the activity of p70S6K and the function of 4E-BP1, leading to inhibition of protein synthesis [68]. Through inhibition of p70S6K, rapamycin also blocks the translation of 5′-TOP (5′-terminal oligopyrimidine tract) mRNAs to suppress mRNA translation and protein synthesis [69]. In addition to affecting p70S6K, 4E-BP1 is a translation inhibitor, which is phosphorylated and inactivated in response to a growth signal [70]. In fact, rapamycin treatment causes the dephosphorylated 4E-BP1 to bind and inhibit the translation initiation factor eIF4E, which then dissociates the cap structure at the 5′ termini of mRNAs, thereby suppressing cap-dependent translation [71]. Thus, inhibiting the mTOR signal strongly reduces intracellular protein synthesis and alleviates the protein load in the ER which, in turn, remarkably suppresses ER stress. In addition, the mTOR signal can directly influence ER stress through a specific Ire1α–ASK1–JNK signal. Inhibiting mTORC1 blocks the Ire1α–ASK1–JNK pathway and suppresses the activation of JNK, which mitigates ER-stress-induced apoptosis [72].

Inhibition of the mTOR signal augments the autophagy process to remove damaged macromolecules and misfolded proteins. Inhibition of mTORC1 leads to increased ULK1/2 (UNC-5 like autophagy activating kinase 1/2) activity, which further phosphorylates *ATG13* (autophagy related gene 13) to activate the autophagy process [73,74]. At the transcriptional level, inhibition of mTORC1 also modulates autophagy through regulating the localization of TFEB (transcription factor EB), an important autophagy gene regulator. Many studies have demonstrated that mTOR inhibition strongly induces autophagy in various model systems, even in the presence of nutrients [75].

Inhibition of mTOR signal by rapamycin and its analogs leads to a reduction in the synthesis of misfolded proteins and an increase in the degradation of damaged proteins, which further suppresses the ER stress caused by gene mutations in cases of retinal degeneration. Many previous studies have demonstrated that inhibition of mTOR has neuroprotective effects, including rescue of photoreceptors and or RPE from mutant gene-induced apoptosis, which slow down the process of retinal degeneration [76].

P23H, one of the Rh mutations, misfolds and accumulates in the ER. If degradation fails, the protein can aggregate to further trigger photoreceptor death. Moreover, mutantP23H negatively competes with the function of the wild-type (WT) protein. However, the toxic and negative properties of mutantP23Hcan beremarkablyattenuated by mTOR inhibition, since P23H aggregation, caspase activation, and apoptosis have been found to be significantly reduced by mTOR inhibition [77]. In an animal model, the activation of UPR and a consistent increase in BiP and CHOP gene expression was detected in P23H Rh retinas with autosomal dominant retinitis pigmentosa (ADRP), which might stimulate apoptotic signaling in these animals. Injections of rapamycin protected the P23H Rh rod photoreceptors from experiencing physiological decline and slowed the rate of retinal degeneration [78].

In P37H mutant retina, chronic suppression of TOR signaling, using the inhibitor rapamycin, was found to strongly mitigate photoreceptor degeneration and chronic P37H proteotoxic stress [79]. Genetic inhibition of the ER stress-induced JNK/TRAF1 pathway and the APAF-1/caspase-9 pathway dramatically suppressed P37H-induced photoreceptor degeneration. These findings suggest that chronic P37H proteotoxic stress disrupts cellular proteostasis, further leading to metabolic imbalance and mitochondrial failure. Inhibiting the mTOR signal can normalize metabolic function and alleviate ER stress-induced retinal degeneration.

RPE dysfunction has been implicated in various retinal degenerative diseases. Impairment of RPE mitochondria in mice induces gradual epithelium dedifferentiation, loss of RPE features, and cellular hypertrophy through activation of the AKT/mTOR pathway. Treatment with rapamycin has been shown to mitigate key features of dedifferentiation and maintain photoreceptor function [77]. However, Rajala et al. reported that light damage caused the activation of the phosphoinositide 3-kinase and the AKT pathway in rod photoreceptor cells. AKT activation, especially the AKT2 signal, plays a neuroprotective role in light-induced retinal degeneration. AKT2 knock-out mice exhibited a significantly greater sensitivity to stress-induced cell death than rods in heterozygous or wild-type mice. These findings suggest that AKT, as a regulator of the mTOR signal, can also function as a therapeutic target when treating retinal degenerative diseases [80,81].

In a rat model of NMDA (*N*-methyl-d-aspartate)-induced retinal neurotoxicity, intravitreal injection of NMDA caused a marked increase of leukocytes and microglia and significant capillary degeneration. However, the NMDA-induced changes were significantly reduced by the simultaneous injection of rapamycin. These findings indicate that mTOR inhibition prevents inflammation and capillary degeneration during retinal injury [82].

**Figure 3:**
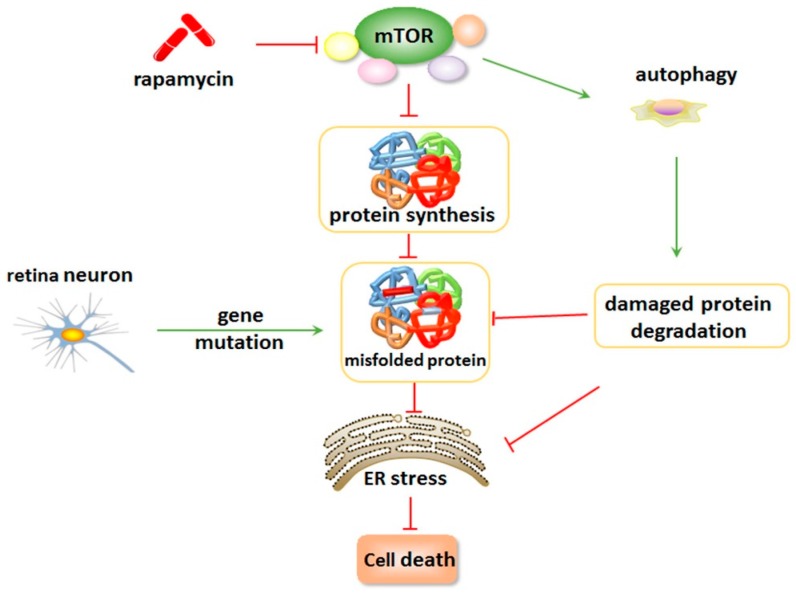
Inhibition of the mTOR signal suppresses ER stress-induced cell death. Inhibition of mTOR maintains cellular proteostasis and attenuates ER stress by reducing misfolded protein synthesis. In addition, inhibition of mTOR can augment autophagy to remove misfolded proteins generated from mutant genes. mTOR inhibition can suppress ER stress-induced apoptosis by regulating retina protein biosynthesis and degradation.

Inhibition of mTOR maintains cellular proteostasis and attenuates ER stress by reducing misfolded protein synthesis and augmenting autophagy to remove misfolded proteins with gene mutations. The mTOR pathway plays an exquisitely complex role in the regulation of retina protein biosynthesis and degradation, as well as in ER stress-induced apoptosis (Figure 3).

## 6. Conclusions

In summary, UPR and ER stress are critical factors that contribute to retinal degeneration, yet inhibition of mTOR maintains cellular proteostasis and attenuates ER stress by reducing misfolded protein synthesis and augmenting autophagy to remove misfolded proteins caused by gene mutations [83]. Further studies are needed to investigate the detailed regulatory network, such as the sensitive surveillance mechanisms of mTORC1 and ER stress, and ER-related apoptosis in retinal neurons. Although the exact mechanism(s) that lead to rapamycin’s neuroprotective effects are unclear, it is tempting to speculate that reduction of misfolded protein synthesis and induction of autophagy help prevent the accumulation of abnormal proteins seen in retinal degenerative disorders, and aids in cell survival in the setting of gene mutation injury. Considering that the field of combined mTOR/UPR research is new, significant progress is likely still ahead.

## Figures and Tables

**Table 1:** Summary of inherited retinal degenerations (IRD) models and dysregulated components.

Animal Model	Mutant Gene	Dysregulated Components	Related Retinal Degeneration	ER Stress Activation	Reference
*Drosophila*	*RhoP23H*	rhodopsin	ADRP	+	[33]
*Xenopus laevis*	*RhoP23H*	rhodopsin	RP	+	[54]
Rats	*RhoP23H*	rhodopsin	RP	+	[50]
Mice	*RhoT17M*	rhodopsin	ADRP	+	[28]
Transfected cell	*ELOVL4*	an enzyme involved in the generation of long-chain fatty acids	Stargardt macular dystrophy	+	[39]
Rd1 mouse	*PDE6-β*	a catalytic subunit of a phosphodiesterase, regulating cGMP levels in photoreceptors	RP	+	[49]
ARPE-19 cells	*R345W*	*N*-Linked glycosylation	Malattia Leventinese and Doyne honeycomb retinal dystrophy	+	[55]

ADRP, autosomal dominant retinitis pigmentosa; RP, retinitis pigmentosa; ER, endoplasmic reticulum.

## Abbreviations

ATF4	Activating transcription factor 4
ASK1	Apoptosis signal-regulating kinase 1
ATF6	Activating transcription factor 6
ATG13	Autophagy related gene 13
AMP	Adenosine monophosphate
AMPK	AMP-dependent kinase
AMD	Age-related macular degeneration
AAV5	Adeno-Associated Virus Type 5
AKT1	AKT serine/threonine kinase 1
adRP	Autosomal dominant RP
BiP	Immunoglobulin binding protein
cGMP	Cyclic guanosine-mono-phosphate
CHOP	C/EBP homologousprotein
cKO	Conditional knockout
DHRD	Doyne honeycomb retinal dystrophy
DEPTOR	Disheveled, Egl-10, and pleckstrin domain-containing mTOR-interacting protein
ERAD	ER-associated protein degradation
ER	Endoplasmic reticulum
ELOVL4	Elongation of very long chain fatty acids
ERK	Extracellular signal related kinase
F3	Fibulin-3
FKBP12	12 kDa Protein FK506-binding protein
GCL	Ganglion cell layer
GRP78	Glucose-regulated protein 78
GAP	GTPase activating protein
HSR	Heat shock response
INL	Inner nuclear layer
IPL	Inner plexiform layer
IRD	Inherited retinal degeneration
IRE1	Inositol-requiring protein 1
Ire1α	Inositol-requiring kinase 1
JNK	c-Jun *N*-terminal kinase
LCA	Leber congenital amaurosis
ML	Malattia Leventinese
mTOR	Mammalian target of rapamycin
mTORC1	mTOR complex 1
mTORC2	mTOR complex 2
mLST8	Mammalian lethal with SEC13 protein 8
mSIN1	Stress-activated MAP kinase-interacting protein 1
ONL	Outer nuclear layer
OS	Outer segment
OPL	Outer plexiform layer
PERK	Protein kinase RNA-like ER kinase
PDE6	Phosphodiesterase
PDK1	3-Phosphoinositide-dependent protein kinase 1
PIKK	PI3K-kinase-related kinase
PI3K	Phosphoinositide 3-kinase
PIP2	Phosphatidylinositol 4,5-bisphosphate
PIP3	Phosphatidylinositol 3,4,5-triphosphate
PRAS40	40 kDa Pro-rich AKT1 substrate 1
PRR5	Pro-rich protein 5
PTEN	Phosphatase and tensin homolog deleted on chromosome 10
PN	Photoreceptor neuron
p90-RSK	Ribosomal S6 kinase
p70S6K	40S ribosomal protein S6 kinase
Rheb	Ras homolog enriched in brain
RICTOR	Rapamycin-insensitive companion of mTOR
Rh	Rhodopsin
RP	Retinitis pigmentosa
RPE	Retinal pigment epithelium
RAPTOR	Regulatory associated protein of mTOR
S6K1	S6 kinase 1
TSC	Tuberous sclerosis complex
TFEB	Transcription factor immunoglobulin E box-binding proteins
TOR	Target of rapamycin
T17M	Threonine-to-methionine mutation at the 17th residue of rhodopsin
UPS	The ubiquitin-proteasome system
UPR	The unfolded protein response
ULK	UNC-5 like autophagy activating kinase
VEGF	Vascular endothelial growth factor
WT	Wild-type
4E-BP1	4E-binding protein 1
5′-TOP	5′-Terminal oligopyrimidine tract
